# The effectiveness and safety of Naoxintong capsules in the treatment of vascular dementia: A protocol for systematic review and meta-analysis

**DOI:** 10.1097/MD.0000000000027930

**Published:** 2021-11-24

**Authors:** Yan-Lin Li, Fang Cheng, Yan Chen, Jun Wang, Zeng-Dong Xiao, Ying Han

**Affiliations:** a Department of Pharmacy, Haibin People's Hospital of Binhai New Area, Binhai New Area, Tianjin, China; b Department of Traditional Chinese Medicine, Haibin People's Hospital of Binhai New Area, Tianjin, China; c Department of Endocrinology, Haibin People's Hospital of Binhai New Area, Binhai New Area, Tianjin, China.

**Keywords:** meta-analysis, Naoxintong capsules, protocol, traditional Chinese medicine, vascular dementia

## Abstract

**Background:**

Traditional Chinese medicine Naoxintong capsules have achieved good results in the treatment of vascular dementia, but there is no evidence-based medical evidence on the effectiveness and safety of the drug. Therefore, this study uses meta-analysis method to systematically evaluate the effectiveness and safety of Naoxintong capsules in the treatment of vascular dementia, with the aim of providing scientific guidance for clinical treatment and practice.

**Methods:**

This study retrieves a total of 7 network electronic databases, including 4 Chinese databases: China biomedical literature database, CNKI, Chongqing VIP database and WANFANG database, and three English databases: PubMed, Embase, The Cochrane Library. Using the combination of theme words and key words to retrieve the Chinese and English database, the literature is searched from January 1, 1990 to October 1, 2021. Two researchers independently sift through the literature, extract data and evaluate the bias risk included in the study, and in the event of a disagreement, the third researcher is invited to discuss the decision, followed by meta-analysis using software RveMan 5.3 and Stata 12.0.

**Results:**

All findings of this study will be published in a peer-reviewed, high-quality academic journal of medicine.

**Conclusion:**

The results of this study will provide evidence for clinicians to find effective and safe methods of treating vascular dementia in TCM.

**OSF registration number:**

DOI 10.17605/OSF.IO/YVF72, https://osf.io/yvf72.

## 1 Introduction

Vascular dementia (VaD) is a clinically common type of dementia, which refers to severe acquired intellectual impairment syndrome caused by various cerebral hypoperfusion cerebrovascular diseases.^[1]^ Patients with VaD usually show severe dysfunction, memory, orientation, attention, language and other abilities decline significantly. It may be accompanied by a certain degree of mental behavior abnormalities or mood disorders, while neurological dysfunction associated with cerebrovascular disease, which is a common type of dementia.^[2,3]^ According to international epidemiological surveys, there are more than 40 million people with dementia worldwide in 2016, and the number continues to rise, with an estimated increase to 115 million in 30 years. Within Asia, VaD has the lowest prevalence of about 0.6% in people over 65 years of age, up to 4.2%, accounting for 15% to 40% of all dementia, and it is the second largest dementia disease after Alzheimer disease.^[4–6]^ However, there is no clear standard therapy for improving cognitive function in Western medicine, mainly referring to Alzheimer disease's therapeutic drugs. Currently the main drugs used in clinical treatment of dementia are cholinesterase inhibitors, brain blood circulation and brain metabolism improvers, and other drugs such as non-retained anti-inflammatory drugs, estrogen and calcium antagonists.^[7–9]^ However, the effectiveness of delaying cognitive decline is not significant.

In recent years, traditional Chinese medicine (TCM) has been used in clinical treatment of VaD, which has played an effect in suppressing vascular inflammatory response, reducing vascular damage and antagonistic apoptosis. TCM Naoxintong capsules (NXTC) is a combination of astragalus, leech, salvia, myrrh, frankincense and other TCM preparations, which has been widely used in the treatment of VaD.^[10,11]^ Because the sample size of current study is too small, the quality of the study is uneven, the results of inconsistent situations occur from time to time. Therefore, it is very necessary to systematically evaluate the effectiveness and safety of NXTC in the treatment VaD by the method of meta-analysis, and provide scientific guidance for clinical treatment and practice.

## 2 Methods

### 2.1 Registration and ethical approval information

#### 2.1.1 Registration information

This protocol has been registered on the OSF (Registration number: DOI

10.17605/OSF.IO/YVF72, registered website: https://osf.io/yvf72).

#### 2.1.2 Ethics and dissemination

The data used in the study are derived from published articles and Internet resources, and no new research involving humans or animals has been carried out, so ethical reviews do not apply to this type of study.

### 2.2 Eligibility criteria

#### 2.2.1 Study types

A comprehensive collection of randomized controlled trials of NXTC in the treatment of VaD is conducted. Regardless of whether or not the random allocation method is described, whether or not the blind method is applied. The language is restricted to Chinese or English.

#### 2.2.2 Participants

All selected patients are laboratory tested and diagnosed with VaD, with no restrictions on the age, gender, race, scope of the disease and education.

#### 2.2.3 Inclusion criteria

All patients meet the diagnostic criteria for VaD.

CT and MRI examinations found a lesions of brain infarction.

Age 40 to 80 years old.

There are cognitive impairments, simple mental state checking scale (MMSE) score 10 to 26 points.

#### 2.2.4 Exclusion criteria

Nonvascular dementia caused by other causes, such as Alzheimer disease and senile dementia.

Patients with severe heart, liver and kidney diseases.

Allergies or allergic reactions to medications.

The types of research are review, conference papers, meta-analysis and animal experiments.

#### 2.2.5 Interventions

The control group is given only conventional Western medicine.

The study group gives NXTC, which can be used alone or in combination with the control group in line with Western medicine treatment. All treatment methods must have a clear method of administration, frequency of administration, dose and course of treatment. Both the control group and the researchers are able to treat underlying diseases such as hypertension, diabetes, hyperlipidemia, cerebral infarction and coronary heart disease.

### 2.3 Types of outcomes

#### 2.3.1 Primary outcomes

1. Mini-mental state examination (MMSE).
2. Montreal cognitive assessment (MoCA).
3. Hasegawa dementia scale (HDS).

#### 2.3.2 Additional outcomes

1. Daily life self-care ability scale (ADL).
2. Blessed behavior scale (BBS) score.
3. Cerebral hemodynamic indicators, including anterior cerebral artery (ACA), middle cerebral artery (MCA), posterior cerebral artery (PCA) blood flow velocity.
4. The incidence of adverse reactions.

### 2.4 Search strategy

This study retrieves a total of 7 network electronic databases, including 4 Chinese databases: China biomedical literature database, CNKI, Chongqing VIP database and WANFANG database, and 3 English databases: PubMed, Embase, the Cochrane Library. The literature is searched from January 1, 1990 to October 1, 2021. At the same time, it is assisted by manual retrieval to retrieve relevant historical materials and TCM classics. Table 1 shows our complete retrieval strategy for literature retrieval using Cochrane Library.

**Table 1:** Retrieval strategy of literature retrieval and collection in Cochrane Library.

Serial number	Search strategy
#1	(Naoxintong capsules):ti, ab, kw
#2	(Dementias, Vascular):ti, ab, kw OR (Vascular Dementias):ti, ab, kw OR (Vascular Dementia):ti, ab, kw OR (Vascular Dementia, Acute Onset):ti, ab, kw OR (Acute Onset Vascular Dementia):ti, ab, kw OR (Subcortical Vascular Dementia):ti, ab, kw OR (Dementia, Subcortical Vascular):ti, ab, kw OR (Dementias, Subcortical Vascular):ti, ab, kw OR (Subcortical Vascular Dementias):ti, ab, kw OR (Vascular Dementia, Subcortical):ti, ab, kw OR (Vascular Dementias, Subcortical):ti, ab, kw OR (Arteriosclerotic Dementia):ti, ab, kw OR (Arteriosclerotic Dementias):ti, ab,kw OR (Dementia, Arteriosclerotic):ti, ab, kw OR (Dementias, Arteriosclerotic):ti, ab, kw OR (Binswanger Disease):ti, ab, kw OR (Disease, Binswanger):ti, ab, kw OR (Chronic Progressive Subcortical Encephalopathy):ti, ab, kw OR (Binswanger Encephalopathy):ti, ab, kw OR (Leukoencephalopathy, Subcortical):ti,ab,kw OR (Leukoencephalopathies, Subcortical):ti, ab, kw OR (Subcortical Leukoencephalopathies):ti, ab, kw OR (Encephalopathy, Subcortical Arteriosclerotic):ti, ab, kw OR (Binswanger's Disease):ti,ab,kw OR (Binswangers Disease):ti, ab, kw OR (Disease, Binswanger's):ti, ab, kw OR (Encephalopathy, Subcortical, Chronic Progressive):ti,ab,kw OR (Subcortical Encephalopathy, Chronic Progressive):ti, ab, kw OR (Subcortical Leukoencephalopathy):ti, ab, kw OR (Subcortical Arteriosclerotic Encephalopathy):ti, ab, kw OR (Arteriosclerotic Encephalopathy, Subcortical):ti, ab, kw OR (Arteriosclerotic Encephalopathies, Subcortical):ti, ab, kw OR (Encephalopathies, Subcortical Arteriosclerotic):ti,ab,kw OR (Subcortical Arteriosclerotic Encephalopathies):ti, ab, kw OR (Encephalopathy, Binswanger's):ti, ab, kw OR (Binswanger's Encephalopathy):ti, ab, kw OR (Encephalopathy, Binswangers):ti, ab, kw OR (Encephalopathy, Binswanger):ti, ab, kw OR (Encephalopathy, Chronic Progressive Subcortical):ti, ab, kw
#3	(Randomized controlled trial):ti, ab, kw
#4	#1 AND #2 AND #3

### 2.5 Research collection and data analysis

**Figure 1:**
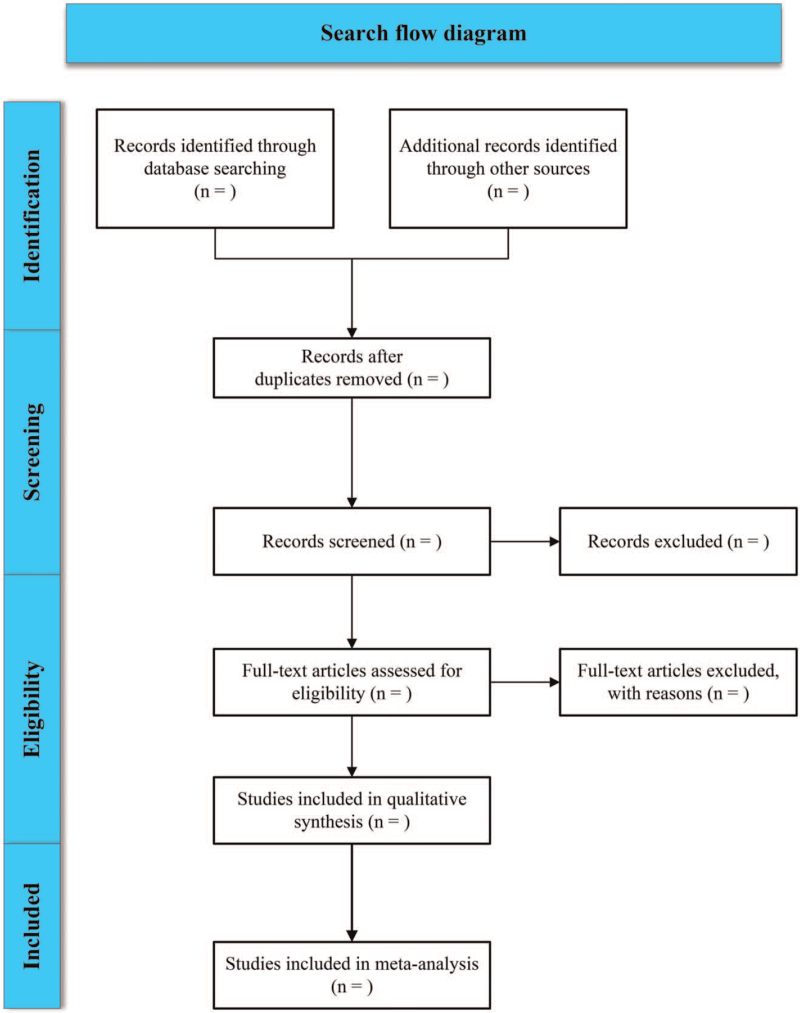
The complete process of literature collecting and screening.

The 2 members conduct separate searches according to the retrieval strategy. Cross-checking the screening results of the 2 members, and if there is a disagreement, the parties will negotiate a settlement or seek the assistance of a third member to determine. The process of collecting and screening literature is shown in Figure 1.

### 2.6 Assessment of risk of bias

This study comprehensively assesses the bias risk in randomized controlled trials using the quality evaluation standard Risk of bias table in Cochrane Handbook 5.1.0, prepared by the Cochrane Collaboration Network, recommended by the International Alliance for Evidence-Based Medicine. This includes 6 aspects, each entry lists three possible scenarios, named low risk, unclear risk, and high risk.

### 2.7 Data synthesis and analysis

The literature included are combined with Review manager 5.3 software, and the total results are presented in forest maps. *I*^2^ statistics and Q statistics are used to test the heterogeneity between the included studies. When *I*^2^ < 50% and Q-test *P* ≥ .05, the heterogeneity among the included studies was small, and the fixed effect model was selected to analyze the results; When *I*^2^ ≥ 50% and Q-test *P* < .05, the heterogeneity among the included studies was large, at this time, if the reasons for the heterogeneity cannot be analyzed and excluded, the random effects model is selected for the result analysis.

### 2.8 Sensitivity analysis

This study will take the following method to analyze the sensitivity of the included research results: if there is homogeneity between the study data, the use of different statistical methods to reanalysis the data, that is, the use of random effect model instead of fixed effect model.^[12]^

### 2.9 Subgroup analysis

For the heterogeneous outcome indicators in this study, subgroup analysis will be conducted from the aspects of drug type, patient's course of disease, administration method, administration frequency, dosage and course of treatment.

### 2.10 Publication bias

This study judges the size of publication bias by analyzing whether the funnel chart is symmetrical and the degree of symmetry.

## 3 Discussion

Modern medicine has not fully clarified the pathogenesis of VaD, the cause is complex, and there are a variety of pathological factors involved in the progression of the disease, such as lipid metabolism disorders, increased blood viscosity, oxidative stress injury and inflammatory cytokine stimulation.^[13]^ At present, the clinical use of choline enzyme inhibitors and antioxidant drugs for treatment, but the effect is not ideal. Studies have shown that the NXTC inhibits nerve cell apoptosis and protects nerve function, while it promotes cerebrovascular dilation, improves blood supply to the brain, regulates microcirculation, promotes oxygen delivery, and relieves hypoxia, thereby improving nerve function in patients.^[14]^ In addition, the tanshinone component contained in the drug has the effects of antagonizing the proliferation of glial cells and reducing the damage of nerve function.^[15]^ Although NXTC have been widely used by clinicians in the treatment of VaD, its effectiveness and safety are still controversial. Therefore, in order to explore the effectiveness and safety of NXTC in the treatment of VaD, this study systematically evaluates the literature, uses the results of the literature to carry out clinical trials, promotes the transformation of evidence into clinical practice, complements and perfects the treatment method of VaD from the direction of Chinese medicine, and it will play a positive role in improving the therapeutic effect of associated dementia.

## Author contributions

**Conceptualization:** Yan-Lin Li, Fang Cheng, Ying Han.

**Data curation:** Yan-Lin Li, Fang Cheng.

**Formal analysis:** Yan-Lin Li, Fang Cheng, Yan Chen, Jun Wang.

**Funding acquisition:** Ying Han.

**Investigation:** Yan-Lin Li, Fang Cheng, Yan Chen, Jun Wang, Zeng-Dong Xiao.

**Methodology:** Yan-Lin Li, Fang Cheng.

**Resources:** Yan-Lin Li, Yan Chen, Zeng-Dong Xiao.

**Software:** Fang Cheng, Yan Chen, Jun Wang.

**Supervision:** Yan-Lin Li, Ying Han.

**Writing – original draft:** Yan-Lin Li, Fang Cheng, Yan Chen, Jun Wang, Zeng-Dong Xiao.

**Writing – review & editing:** Ying Han.
